# Effect of solvents on bioactive compounds and antioxidant activity of *Padina tetrastromatica* and *Gracilaria tenuistipitata* seaweeds collected from Bangladesh

**DOI:** 10.1038/s41598-021-98461-3

**Published:** 2021-09-27

**Authors:** Mohammad Khairul Alam Sobuj, Md. Ariful Islam, Md. Shoebul Islam, Md. Mohidul Islam, Yahia Mahmud, S. M. Rafiquzzaman

**Affiliations:** 1 Marine Fisheries and Technology Station, Bangladesh Fisheries Research Institute, Cox’s Bazar, 4700 Bangladesh; 2 Department of Fisheries Biology and Aquatic Environment, BSMRAU, Gazipur, 1706 Bangladesh; 3 Bangladesh Fisheries Research Institute, Mymensingh, 2201 Bangladesh

**Keywords:** Biochemistry, Chemical biology, Drug discovery

## Abstract

Seaweeds are now recognized as a treasure of bioactive compounds. However, the bioactivity of seaweed originating in Bangladesh is still unexplored. So, this study was designed to explore the secondary metabolites and antioxidant activities of solvent extracts of *Padina tetrastromatica* and *Gracilaria tenuistipitata*. Phytochemical screening and FTIR spectra confirm the diverse type of bioactive compounds. Antioxidant activity of extracts were evaluated by 1,1-diphenyl-2-picrylhydrazyl (DPPH), 2, 2-Azino-bis (3-ethylbenzothiazoline-6-sulfonic acid) (ABTS), reducing power (RP), phosphomolybdenum, hydrogen peroxide and nitric oxide (NO) scavenging assays. Here, methanolic extract of *P. tetrastromatica* showed highest amount of total phenolic content (85.61 mg of GA/g), total flavonoid content (41.77 mg of quercetin/g), DPPH (77.07%), ABTS (77.65%), RP (53.24 mg AAE/g), phosphomolybdenum (31.58 mg AAE/g), hydrogen peroxide (67.89%) and NO (70.64%) assays compared to its methanolic extracts of *G. tenuistipitata*. This study concluded that methanol as a solvent extract of brown seaweed (*P. tetrastromatica*) exhibited bioactivity and antioxidant potentiality which will be useful for pharmacological as well as in functional food application.

## Introduction

Free radicals are chemical species (atoms, molecules, or ions) that are incredibly reactive, usually contain unpaired electrons, and can be produced in living cells from endogenous or exogenous sources^1^. Endogenous free radicals are formed during metabolism due to multiple biochemical reactions inside the cell^2^, whereas exogenous stimulants of free radical production include pollutants, heavy metals, tobacco, smoke, drugs, xenobiotic, and radiation^3^. Examples of free radicals include peroxides (O_2_^2−^), peroxynitrite (ONOO^−^), superoxide (·O_2_^−^), hydroxyl radical (·OH), alpha-oxygen (α-O), nitric oxide (NO), hydrogen peroxide (H_2_O_2_), nitrogen dioxide (NO_2_), and singlet oxygen (^1^O_2_), etc. The presence of free radicals will result in several damages like denaturing of enzymes and cellular proteins, lipid peroxidation in tissue membranes, nucleic acid disruption, and cellular function distraction^3,4^. These damages by free radicals are termed oxidative stress, which is reported to be responsible for various diseases such as ADHD^5^, autism^6^, cancer^7^, Alzheimer’s disease^8^, Parkinson's disease^9^, and aging^10^. Hence, removing free radicals from our body is the only ultimate concerning approaches to protect in contradiction of these diseases.

Antioxidants are organic compounds that can neutralize the body’s excess free radicals and protect cellular structures such as DNA, proteins, and lipids from oxidative damage^11^. Our bodies should always have the ability to maintain equilibrium between free radical development and antioxidant availability to prevent cell damage. To retain the oxidant-antioxidant stability in control, it is essential to supply a sufficient amount of antioxidants in the body through diet. In this case, natural sources of antioxidants such as ascorbic acid, chlorophyll derivatives, polyphenols, amines, amino acids, and flavonoids might be more efficient than synthetic antioxidants. However, due to their carcinogenicity and health effects, prohibitions on the use of synthetic antioxidants, including butylated hydroxytoluene (BHT) and butylated hydroxyanisole (BHA), are being implemented^12^. In recent years, experts are centering on looking at the characteristic sources of natural antioxidant-rich diet materials instead of synthetic ones. Seaweeds have recently been discovered to be an amusing source of bioactive natural compounds with potential antioxidant activities^13–16^.

Seaweeds are microscopic and primarily macroscopic, multicellular, polyphyletic and photosynthetic marine algae and usually grow on the seabed between the coastal region's high tide and low tide zones. Marine algae or seaweeds mostly grow in the rocky part of the littoral zone of the ocean, where usually 0.01% light penetration can assist the photosynthesis of the seaweeds^17^. The maximum significant application of seaweed is found in food industries, cosmetic industries, an industry of phycocolloid or hydrocolloid, biofuel production, wastewater treatment, pharmaceutical industry and fertilizer^18,19^. In general, seaweed contains different secondary metabolites, for example, tannins, saponins, phenols, and flavonoids in varying concentration^16,20^. In addition, seaweed has a wide range of bioactive compounds that have antibacterial, anti-Alzheimer’s, anti-inflammatory, antifungal, anti-hyperglycemic, anti-aging activities, and preferable antioxidant properties^21–24^. Seaweed diversity is vibrant on the Bangladesh coast and described that there are approximately 193 algal species, of which 51 Chlorophyta (green), 54 Phaeophyta (brown), and 88 are Rhodophyta (red) class occurring on Bangladesh coastline^25^.

Among various seaweeds found in the Saint Martin’s Island, northeastern part of Bay of Bengal, Bangladesh region, *P. tetrastromatica* and *G. tenuistipitata* are abundantly available in this diverse ecosystem. However, investigations into recognizing seaweed assets in Bangladesh are ineffectively evoked, although it has enormous possibilities. Although several studies across the world have demonstrated the antioxidant capabilities of seaweed in the last two decades, there is scanty information regarding the antioxidant potential and bioactivity of these two species grown in Bangladesh. Previous researchers typically only investigated the proximate biochemical and nutritional analysis of the seaweed^26,27^. There is limited evidence in the literature regarding the bioactivity and antioxidant properties of seaweed obtained off the coast of Bangladesh^16,28^. This type of research is a primary step towards validating a seaweed species as an important commercial species. Additionally, this research facilitates the discovery of novel marine drugs by using specific solvents from the seaweed resources of Bangladesh. Furthermore, the possible application of both the seaweeds as potent sources of natural antioxidants as food supplements or functional feed will be evaluated. A deeper understanding of this concept is required as the bioactivity, and chemical composition of seaweed vary depending on geographic location and species variations^29^. Hence, in the current study, we used a variety of qualitative and quantitative tests (Phytochemical analysis and FTIR) to screen and measure for functionally bioactive compounds and determine antioxidant activities using various in vitro spectroscopic assays, as well as their correlation among different assays of various crude extracts of *P. tetrastromatica* and *G. tenuistipitata*.

## Results

### Phytochemical screening

The six crude extracts for two seaweed species were screened for the occurrence of six phytochemicals named saponin, terpenoid, cardiac glycoside, phlobatannin, phenolic, and flavonoid. 29 of the 36 tested samples were positive, while the other seven were negative. It was observed that every extract contained varying amounts of active secondary metabolites (phytochemicals) such as saponin, terpenoid, cardiac glycoside, phlobatannin, phenolic and flavonoid (Table 1).

**Table 1:** Preliminary phytochemical screening of different extracts of *P. tetrastromatica* and *G. tenuistipitata*.

Seaweed species	Extracts	Saponin	Terpenoid	Cardiac glycoside	Phlobatannin	Phenolic	Flavonoid
*P. tetrastromatica*	Methanol	$+$	$+$	$+$	$+$	$+$	$+$
	Ethanol	$+$	$-$	$+$	$-$	$+$	$+$
	Water	$+$	$-$	$-$	$+$	$+$	$+$
*G. tenuistipitata*	Methanol	$+$	$+$	$+$	$+$	$+$	$+$
	Ethanol	$-$	$+$	$-$	$+$	$+$	$+$
	Water	$+$	$-$	$+$	$+$	$+$	$+$

“+” constituent's existence, “−” constituent's non-existence.

### FTIR analysis

**Figure 1:**
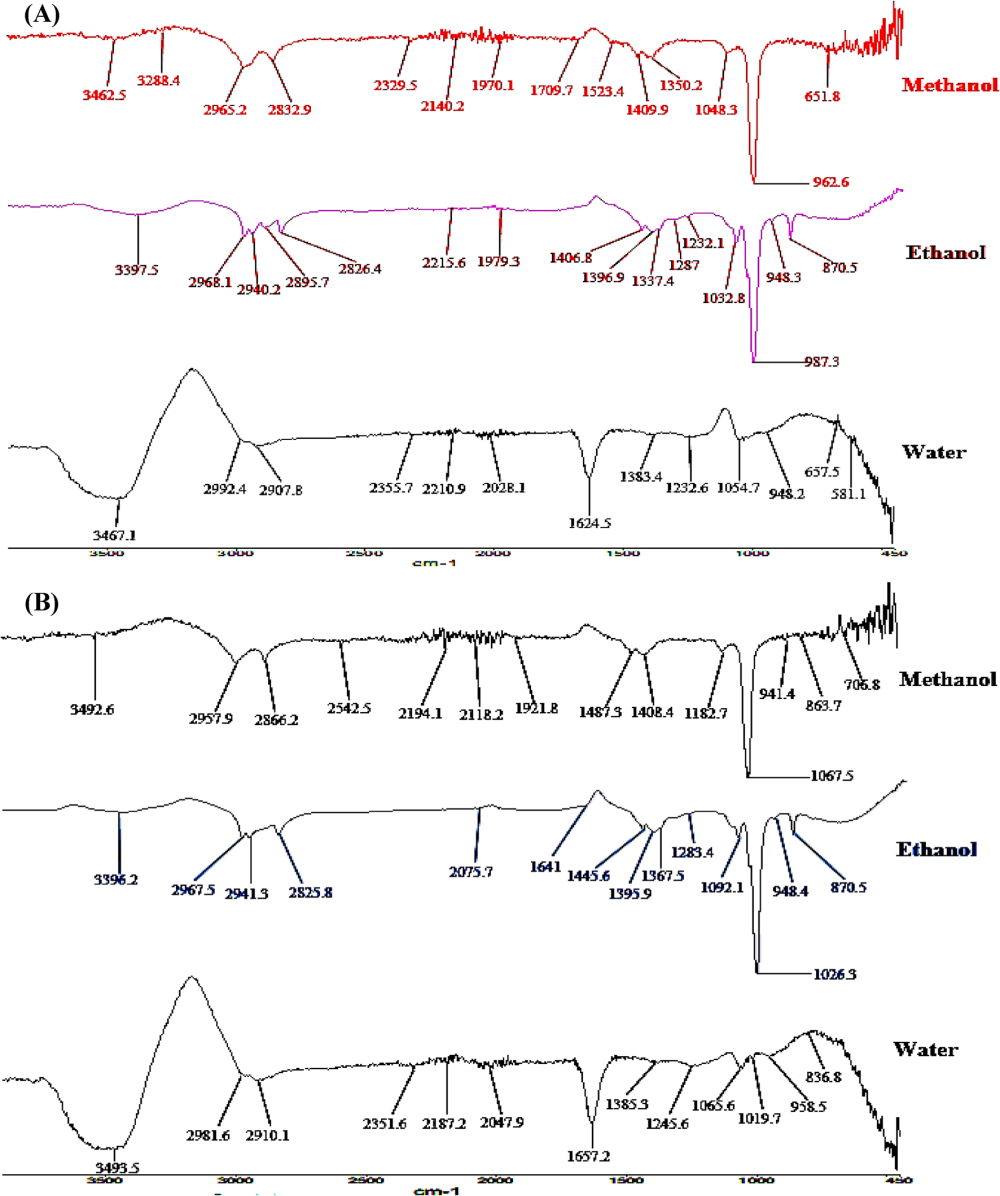
FTIR spectrum of different solvent extracts of seaweed (**A**) *P. tetrastromatica* and (**B**) *G. tenuistipitata*.

Different solvent extracts (methanol, ethanol and water) of *P. tetrastromatica* and *G. tenuistipitata* showed distant peaks that reported various functional groups in the 4000–450 cm^−1^ range. The existence of phenols, carboxylic acids, alkoxy, aromatic, alkene, amides/amines, and sulfonate compounds was verified by the findings of the FTIR study, which ensured the presence of O–H, N–H, C–H, C=O, C–C, C–N and S=O bonds at different extracts (Table 2; Fig. 1). The single bond area (2500–4000 cm^−1^) of seaweed extracts revealed a variety of peaks. The O–H stretch of H-bonded alcohols and phenols causes the peaks at 3493.5, 3492.6, 3467.1, 3426.5, 3396.2, 3397.5 cm^−1^. In the case of *G. tenuistipitata*, the strong pick at 1026.3 and 1067.5 cm^−1^ is due to the C–O stretch of primary alcohol. The existence of the O–H stretch of carboxylic acids is shown by bands in the range of 2700–3300 cm^−1^ (2965.2, 2957.9, 2940.2, 2910.1, 2907.8, 2825.8 cm^−1^). The C–O stretch of alkoxy can be found in the peaks at 1092.1, 1065.6, 1054.7, 1048.3, 1032.8, 1048.3 cm^−1^. In the peaks at 836.8, 863.7, 870.5 cm^−1^, the C–H stretch (aromatics) can be found. The pick at 1657.2 and 1624.5 cm^−1^ in water extracts of both seaweeds was for the C=C stretch of aromatics. The C–H stretch was observed in the alkene peaks at 987.3, 962.6, 958.5, 948.4, 948.2, and 941.4 cm^−1^. In the pick at 1657.2, 1641, 1624.5 cm^−1^, the C=O strip of amide was visible. The sulfonates showed NO_2_ and SO_2_ stretch in the range of 1100–1200 cm^−1^ and S=O stretch near 1300–1365 cm^−1^.

**Table 2:** Major functional groups of active components based on the peak value of Fourier transform infrared.

Seaweed species	Extracts	Major functional groups						
		Phenol	Carboxylic acids	Alkoxy	Aromatics	Alkene	Amides/amines	Sulfonate
*P. tetrastromatica*	Methanol	$+$	$+$	$+$	$-$	$+$	$-$	$+$
	Ethanol	$+$	$+$	$+$	$+$	$+$	$-$	$+$
	Water	$+$	$+$	$+$	$+$	$+$	$+$	$-$
*G. tenuistipitata*	Methanol	$+$	$+$	$+$	$+$	$+$	$-$	$+$
	Ethanol	$+$	$+$	$+$	$+$	$+$	$+$	$-$
	Water	$+$	$+$	$+$	$+$	$+$	$+$	$-$

“+” constituent's existence, “−” constituent's non-existence.

### Quantitative phytochemical analysis

#### Total phenolic content (TPC)

The overall amount of total phenols in different crude extracts was measured using FC reagent and external calibration with Gallic acid at a concentration of 7 mg mL^−1^. TPC levels varied significantly among solvent extracts, ranging between 85.61 and 34.11 mg of GA/g (Table 3). Methanolic extract of *P. tetrastromatica* has the particularly maximum level of TPC (85.61 mg of GA/g), followed by ethanol and water extracts (74.59 and 42.73 mg of GA/g, respectively) (p < 0.05) (Table 3). Additionally, methanol extracts (68.20 mg of GA/g) have the maximum volume of TPC of *G. tenuistipitata*, followed by ethanol extract (61.65 mg of GA/g) and water extract (34.11 mg of GA/g) (p < 0.05) (Table 3).

**Table 3:** Total phenolic and flavonoid content of *P. tetrastromatica* and *G. tenuistipitata* crude extracts.

Extracts	*P. tetrastromatica*		*G. tenuistipitata*	
	Total phenolics (mg of GA/g)	Total flavonoids (mg of quercetin/g)	Total phenolics (mg of GA/g)	Total flavonoids (mg of quercetin/g)
Methanol	85.61 ± 1.47^a^	41.77 ± 1.59^a^	68.20 ± 0.92^a^	36.17 ± 2.38^a^
Ethanol	74.59 ± 0.70^b^	35.27 ± 1.64^b^	61.65 ± 1.27^b^	26.42 ± 0.90^b^
Water	42.73 ± 2.09^c^	22.39 ± 0.72^c^	34.11 ± 0.88^c^	19.86 ± 1.39^c^

Different superscript letters represent things that are substantially different from one another. Significance level is p < 0.05.

#### Total flavonoid content (TFC)

The aluminum chloride procedure was used to calculate the concentration of total flavonoid content in different crude extracts at a concentration of 7 mg mL^−1^. Methanol extract showed significantly highest amount of TFC in the case of both seaweeds, followed by ethanol and water extracts (p < 0.05) (Table 3).

### Evaluation of total antioxidant capacity

#### DPPH assay

**Figure 2:**
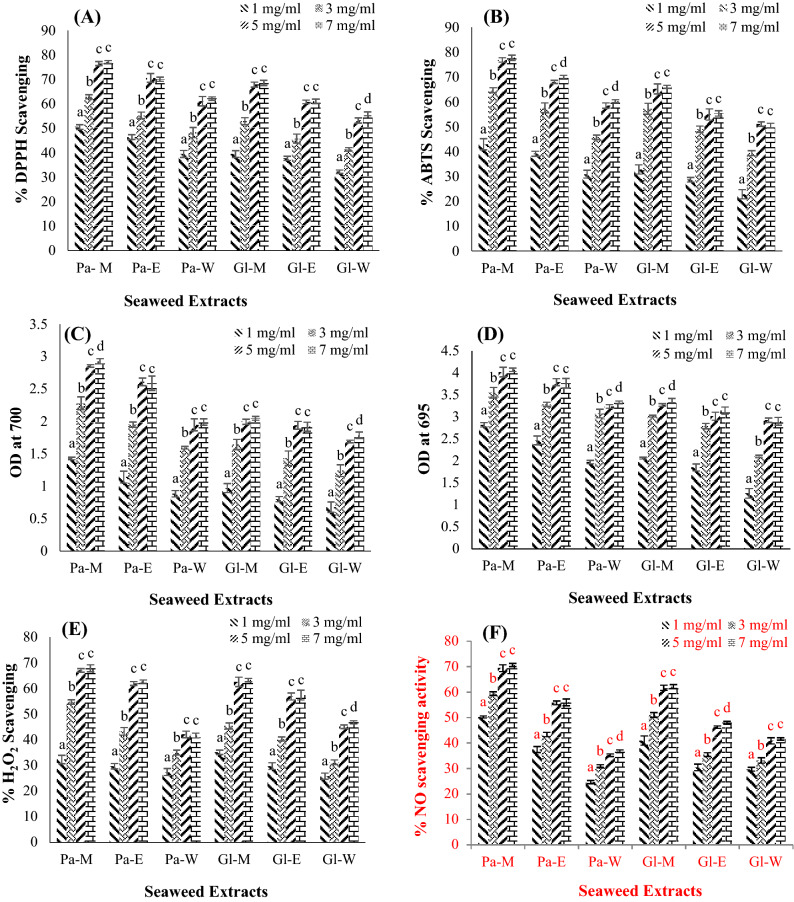
(**A**) 1, 1-diphenyl-2-picrylhydrazyl assay, (**B**) 2, 2-azino-bis (3-ethylbenzothiazoline-6-sulfonic acid) assay, (**C**) Reducing power assay, (**D**) Phosphomolybdenum assay, (**E**) Hydrogen peroxide scavenging assay, **(F)** NO scavenging assay of different crude extracts of *P. tetrastromatica* and *G. tenuistipitata*. Pa—*Padina tetrastromatica*, Gl—*Gracilaria tenuistipitata*, M—methanol, E—ethanol, W—water.

In this process, nitrogen-free radical in the DPPH is readily scavenged by the antioxidant compounds, and the purple color of DPPH solution is cleared by the antioxidants. The findings show that the antioxidant activity of crude seaweed extracts increases dramatically as the concentration of seaweed extract increases (p < 0.05). The percentage of inhibition of methanolic extracts of *P. tetrastromatica* and *G. tenuistipitata* (77.08 and 68.54%, respectively) was slightly higher (p < 0.05) than that of ethanolic and water extracts (Fig. 2A). Compared to the positive control (i.e., ascorbic acid, IC_50_ = 0.00297 mg mL^−1^), the IC_50_ values of all crude extracts showed lower DPPH radical scavenging effects (Table 4).

**Table 4:** Values of IC_50_ (mg mL^−1^) resultant from various crude extracts of various antioxidant assays.

Antioxidant assay	Seaweed species	IC_50_ (mg mL^−1^) values of different crude extracts			
		Ascorbic acid	Methanol	Ethanol	Water
DPPH assay	*P. tetrastromatica*	0.00297 ± 0.0005^a^	0.40 ± 0.12^b^	1.54 ± 0.03^c^	3.31 ± 0.04^d^
	*G. tenuistipitata*		2.59 ± 0.08^b^	3.68 ± 0.07^c^	5.04 ± 0.10^d^
ABTS radical scavenging assay	*P. tetrastromatica*	0.160 ± 0.006^a^	1.33 ± 0.09^b^	2.31 ± 0.06^c^	4.22 ± 0.11^d^
	*G. tenuistipitata*		3.01 ± 0.10^b^	4.69 ± 0.08^c^	5.91 ± 0.03^d^
H_2_O_2_ scavenging assay	*P. tetrastromatica*	0.0783 ± 0.004^a^	3.08 ± 0.05^b^	4.10 ± 0.04^c^	9.41 ± 0.09^d^
	*G. tenuistipitata*		3.69 ± 0.11^b^	4.78 ± 0.05^c^	7.32 ± 0.07^d^
NO scavenging assay	*P. tetrastromatica*	0.0824 ± 0.008^a^	0.75 ± 0.03^b^	4.55 ± 0.05^c^	12.94 ± 0.06^d^
	*G. tenuistipitata*		2.93 ± 0.04^b^	7.16 ± 0.04^c^	10.31 ± 0.03^d^

Different superscript letters represent things that are substantially different from one another. Significance level is p < 0.05.

#### ABTS radical scavenging assay

In vitro antioxidant activity by ABTS radical scavenging assay comprises the reaction that results in the formation of a blue-green ABTS chromophore between ABTS and hydrogen donating oxidizing agent, in this case, potassium persulfate. Among the methanolic extracts, *P. tetrastromatica* recorded significantly higher ABTS free radical scavenging activity (77.65%, IC_50_ = 1.33 mg mL^−1^) followed by *G. tenuistipitata* (66.09%, IC_50_ = 3.01 mg mL^−1^) (Fig. 2B). As shown in Table 4, the IC_50_ values exhibited the order (methanol > ethanol > water), comparable to extracts with phenolic and flavonoid content. Compared to the positive control (i.e., ascorbic acid, IC_50_ = 0.16 mg mL^−1^), the IC_50_ values of all crude extracts showed lower ABTS radical scavenging effects (Table 4).

#### Reducing power assay

The antioxidant activity of altered crude extracts was evaluated using the reducing power assay. This assay is dependent on the hydrogen ion in antioxidants reducing ferric (Fe^3+^) to ferrous (Fe^2+^) product, changing the color of the substance to different shades of green to blue depending on the antioxidant function. Here *P. tetrastromatica* showed significantly higher (p < 0.05) absorbance (A_700nm_ 0.885–2.927) compared to the absorbance of *G. tenuistipitata* (A_700nm_ 0.678–2.047) (Fig. 2C). Ascorbic acid was used as a reference compound to determine the reduction ability of different crude extracts from the seaweed species. The crude methanolic extract had the highest reducing power of all the samples tested of *P. tetrastromatica* and *G. tenuistipitata*, and the data were 53.24 and 46.81 mg of AAE/g, respectively.

#### Phosphomolybdenum assay

To determine the antioxidant ability of extracts, the phosphomolybdenum method is widely utilized. In this method, converting Mo (VI) to Mo (V) forms phosphomolybdenum (V) complex, a bluish-green colored compound in the presence of antioxidant-containing substances. Here *P. tetrastromatica* showed significantly higher (p < 0.05) absorbance (A_695nm_ 4.071) compared to the absorbance of *G. tenuistipitata* (A_695nm_ 3.369) (Fig. 2D). However, in the context of water extract, *G. tenuistipitata* showed better absorbance at 5 mg mL^−1^ (A_695nm_ 2.922) than 7 mg mL^−1^ (A_695nm_ 2.897). In the crude methanolic extract, *P. tetrastromatica* had the highest antioxidant activity of 31.58 mg of AAE/g, while *G. tenuistipitata* had 19.27 mg of AAE/g.

#### Hydrogen peroxide scavenging activity

Methanolic extracts of *P. tetrastromatica* (67.89%) and *G. tenuistipitata* (63.28%) had slightly higher (p < 0.05) scavenging efficacy than ethanol and water extracts (Fig. 2E). Extracts showed their activity in a concentration-dependent manner. As shown in Table 4, the IC_50_ values of H_2_O_2_ scavenging ability exhibited the order (methanol > ethanol > water), comparable to the DPPH and ABTS scavenging activity. When compared to all other crude extracts, the IC_50_ value of the positive control (i.e., ascorbic acid, IC_50_ = 0.0783 mg mL^−1^) indicated a greater propensity to scavenge H_2_O_2_ (Table 4).

#### NO scavenging assay

The antioxidant activity of crude extracts was evaluated using the nitric oxide scavenging assay. Here, methanolic extracts of *P. tetrastromatica* (70.64%) and *G. tenuistipitata* (62.18%) had slightly higher (p < 0.05) scavenging efficacy than ethanol and water extracts (Fig. 2F). The scavenging activity showed concentration-dependent manner for the all the crude extracts of both seaweed. As shown in Table 4, the IC_50_ values of NO scavenging ability exhibited the order (methanol > ethanol > water) which is similar to the DPPH, ABTS and H_2_O_2_ scavenging activity. Compared to the positive control (i.e., ascorbic acid, IC_50_ = 0.0824 mg mL^−1^), the IC_50_ values of all crude extracts showed lower NO scavenging effects (Table 4).

### Correlations analysis

#### Correlation between total phenolic contents and different antioxidant activity assays

**Figure 3:**
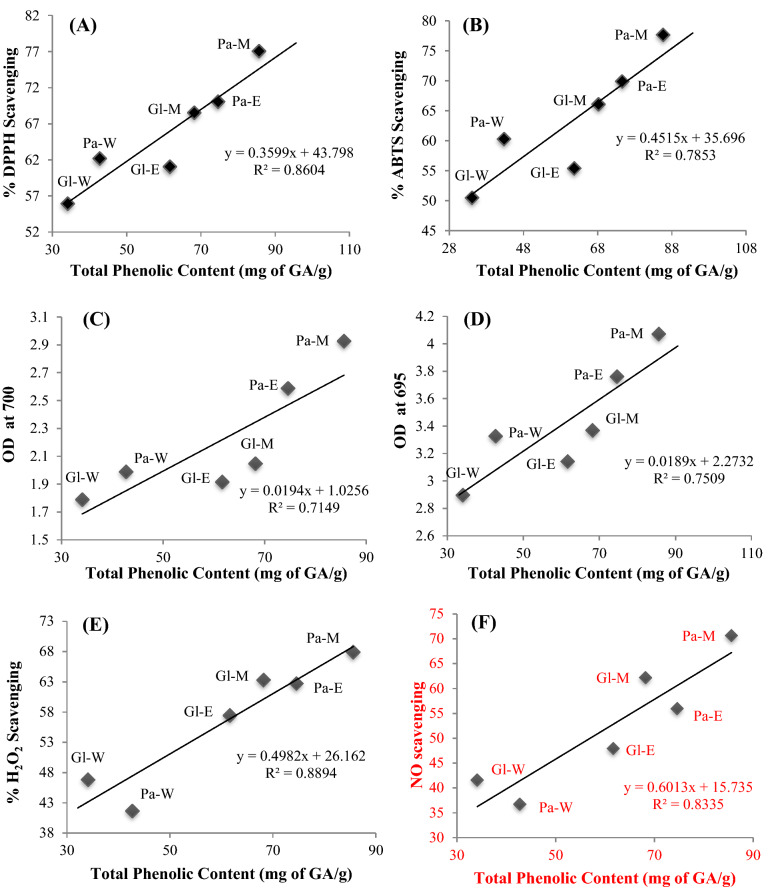

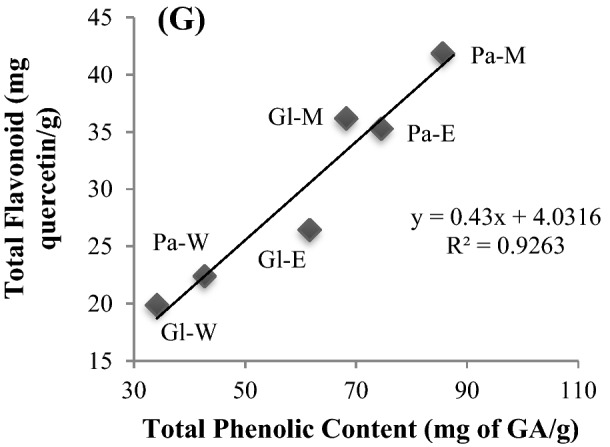
Scatter plot diagrams showing the correlation of total phenolic content (mg of GA/g) vis-à-vis (**A**) DPPH (n = 6; R^2^ = 0.8604), (**B**) ABTS (n = 6, R^2^ = 0.7853), (**C**) Reducing ability (n = 6, R^2^ = 0.7149), (**D**) Phosphomolybdenum (n = 6, R^2^ = 0.7509), (**E**) H_2_O_2_ scavenging activity (n = 6, R^2^ = 0.8894), **(F)** NO scavenging (n = 6, R^2^ = 0.8335) and (**G**) total flavonoid content (n = 6; R^2^ = 0.9263). Pa—*Padina tetrastromatica*, Gl—*Gracilaria tenuistipitata*, M—methanol, E—ethanol, W—water.

The Pearson correlation analysis approach established a strong positive linear correlation between total phenolic contents (TPC) and various radical scavenging assays of seaweed extracts [TPC-DPPH: R^2^ = 0.8604 (Fig. 3A), TPC-ABTS: R^2^ = 0.7853 (Fig. 3B), TPC-reducing ability: R^2^ = 0.7149 (Fig. 3C), TPC-Phosphomolybdenum: R^2^ = 0.7509 (Fig. 3D), TFC-H_2_O_2_ scavenging activity: R^2^ = 0.8894 (Fig. 3E), TFC-NO scavenging: R^2^ = 0.8335 (Fig. 3F)]. All antioxidant activities of crude seaweed extracts were positively correlated with one another, which evidently point out that phenolic compounds are primarily accountable for the antioxidant properties.

#### Correlations between total phenolic contents and total flavonoid contents

The Pearson correlation analysis approach revealed a solid and positive linear correlation between total phenolic content (TPC) and total flavonoid content (TFC) of various seaweed extracts [TPC-TFC: R^2^ = 0.9263 (Fig. 3G)]. Prospective studies show that total phenolic and flavonoid content are significant antioxidant activity determinants in different crude extracts of seaweed.

## Discussion

The physiological and mechanical capabilities of marine living beings that permit them to endure in multifaceted living forms give an extraordinary impending generation of secondary metabolites (phytochemicals), which are not observed in earthborn circumstances. Hence, crude extracts of seaweed are amongst the foremost excessive fountainheads of unique, exceptional, and identified bioactive compounds^30^. And the fact that only a few studies have been conducted on Bangladeshi seaweed assets emphasizing its bioactivity or secondary metabolites existences. Hence, it becomes time demanding to be familiar with almost completely unexplored Bangladeshi seaweed assets for way more excellent knowledge of its bio-functional activity as the abundance and accessibility of bioactive compounds of seaweeds are to a significant extent changes concurring to geographic area, natural condition, season, development and fair as the profundity of inundation^29^. In Bangladesh, however, there is a lack of information on available secondary metabolites and antioxidant properties of seaweed. However, other researchers explored Bangladeshi seaweeds, and a variety of phytochemicals and promising antioxidant properties were discovered^16,28^. Hence, in the present study, we used a combination of qualitative and quantitative tests (Phytochemical analysis and FTIR) to screen out for functionally bioactive compounds and determine antioxidant activities using various in vitro spectroscopic assays of different crude extracts of two significant Bangladeshi seaweeds (*P. tetrastromatica* and *G. tenuistipitata*). Furthermore, a correlation between TPC, TFC, and antioxidant activity was investigated in order to well appreciative the role of phenols and flavonoids in antioxidant activity.

The presence of any phytoconstituents primarily influenced by the dissolvable solvent used for extraction and the seaweed physicochemical properties. The essential bioactive compounds in seaweed can be screened using various methods while keeping different solvents and situations in consideration^28^. In this study, we used methanol, ethanol, and water extracts having a dielectric constant of about 33, 25, and 80, respectively^31^. The phytochemical screening indicates active secondary metabolites such as saponin, terpenoid, cardiac glycoside, phlobatannin, phenolic, and flavonoid in various extracts at different concentrations (Table 1). Among them, terpenoids were absent in the aqueous extract in both seaweed because they are non-polar compounds and required non-polar solvents for extraction^32^. Besides these, all components showed positive results in the methanolic extract of both seaweeds. Our present finding coincides with the findings of other authors; who also found several phytochemicals in case of altered solvents from brown seaweed *P. tetrastromatica*^20,33^*.* But in the case of *G. tenuistipitata* there has been no prior research on preliminary phytochemical screening was found in the literature. However, some scholars also identify several phytoconstituents from red algae *G. corticata* and *G. verrucosa* respectively, which the current results validate^34,35^. As indicated by the relevant studies, several phytochemicals in red algae *H. musciformis* collected in Bangladesh, linked to the current observation^16^.

Fourier transformed infrared spectroscopy (FTIR) can be intended to qualitatively analyze different functional groups in seaweed crude extracts (Fig. 1). FTIR analysis ensured phenols, carboxylic acids, alkoxy, aromatic, alkene, amides/amines, and sulfonates in the crude extracts of seaweed (Table 2). Previous researcher also used FTIR to identify several phytochemicals from brown seaweed *P. tetrastromatica*, associated with our current observation^33,36^. They noticed a different category of compounds in other extracts, which may be attributed to differences in extraction methods and seaweed origin. Also, some scholars identify different functional groups from red seaweed *G. rubra* and *H. musciformis*, respectively, which is almost similar to the present findings^16,37^. The incidence of specific fatty acids in various extracts has been observed, determining each extract's antioxidant activity.

The polarity of any solvent plays a significant role in the extraction of phenolic compounds from some plant or fruit^38^. Since it can suppress polyphenol oxidase activity, methanol is usually the most effective solvent for polyphenolic extraction^39^. Our present study found that methanolic extract contained a significant amount of phenolics, 85.61 mg of GA/g for *P. tetrastromatica* and 68.20 mg of GA/g for *G. tenuistipitata*. In contrast, ethanol and water extract contain fewer amounts (Table 3). TPC’s current observation is underpinned by the findings of other scholars^40–42^, who also reported that methanolic extract when compared to other extracts; extract has the most incredible volume of TPC. Similar results in red and brown seaweed obtained from the Bangladeshi coast^28^. Some academics reported 69.5 and 25.29 mg GAE/g, respectively in the methanolic extracts of *P. tetrastromatica*, which is a more petite figure than the one we have now^43,44^. This wider variety of results may be attributed to environmental conditions, the origin of the seaweed, or the varietal extraction method. Similarly, methanol extract has the most significant percentage of total flavonoid content for both seaweed species (Table 3). Our results obtained are similar to the case of other researchers, who also found that methanolic extracts showed the maximum quantity of TFC compared to other solvents^16,21,43^.

Flavonoids are natural phenolic compounds having a unique structural characteristic which leads them to a wide range of biofunctional properties, like free radical scavenging and antioxidant properties^45^. It is often difficult to quantify the antioxidant efficacy of any natural extracts as individual studies work through unique specific mechanisms^46^. More specifically, different methods such as PTIO and nitroprusside-Griess reagent are being used for performing NO scavenging assay^47^. In our present study, different in vitro antioxidant assays including DPPH, ABTS, reducing power assay, phosphomolybdenum, H_2_O_2_ scavenging and NO scavenging assay were performed to evaluate antioxidative properties of the crude extracts of two seaweeds. The antioxidant efficacy of altered crude extracts increased with increasing concentration, showing that these properties are dose dependent. The influence of the amount of bioactive phytochemicals might be responsible for higher antioxidant activity with the increase of concentration. In the case of all antioxidant assays, it was observed that brown seaweed (*P. tetrastromatica*) showed higher activity compared with the red seaweed (*G. tenuistipitata*). An approach similar to ours has been presented earlier^28,48^. The antioxidant capacity of the crude extracts ordered in the rank methanol, ethanol and water extract (Fig. 2A–F), which is similar with the findings of other researchers^16,49–51^. This is due to the fact that methanol extracts can have an H-donating property, allowing them to stop the oxidation process by transforming free radicals to stable compounds. However, the highest effect was observed for ethyl acetate fraction in the case of *P. pavonica*^52^, ethyl acetate and petroleum ether fraction in the case of *G. verrucosa*^53^ and aqueous extract in the case of *P. boergesenii*^54^ which are contradictory to the present findings. These differences might be due to the variation in solvent used for analysis and the differences in the analytical method. Here, TPC and various antioxidant assays of seaweed extracts shown a strong positive linear correlation. Other scholars also documented a similar positive linear correlation amongst TPC and various antioxidant activities of seaweed extract^16,28,55^. Though, other researcher observed a negative correlation between TPC and antioxidant activity (activity of lipid peroxidation inhibition) in the case of red seaweed which test was not performed in our current research^56^. Furthermore, total phenolics and flavonoid content of various seaweed extracts had a similar positive correlation which is in agreement with the results of other researchers^28,57^. Our present finding evidently recommends the existence of phenolic or flavonoid compounds in methanolic extracts may be primarily responsible for the result of the highest antioxidant activity in the crude extracts.

## Methods

### Seaweed sample collection and preparation

Mature *G. tenuistipitata* and *P. tetrastromatica* sample were collected from the wild source at Saint Martin’s Island (92° 28′ 40.12″ E and 20° 65′ 51.43″ N) of Bay of Bengal of Bangladesh in March 2020. Saint Martin's Island is still considered a biologically diverse ecosystem free of external pollutants, with a dense growth of various seaweeds. Permission of sample collection was gained from the local government before harvesting seaweed following local and national regulations. In this experiment, samples of two different species of seaweeds (one red and one brown) were commonly found in the rocky surfaces during low tide. Dr. Md. Enamul Hoq, Former Director of BFRI, authenticated the botanical identification of seaweed species as the voucher specimen has been previously deposited at BFRI herbarium [BFRI (MFTS-RS-18/19-034) and BFRI (MFTS-BS-18/19-048)]. The entire plant was collected from the exposed rock to ensure that the holdfast would not be left out. The collected thallus was washed thoroughly with clean seawater to remove dirt, sand, and other impurities. The specimen was preserved in an icebox at 4 ℃ and transported to the laboratory to maintain the fresh quality. Fresh samples were then washed thoroughly with distilled water for further removal of any other remaining impurities. Cleaned seaweed was then kept in a freeze dryer (VaCo 2, Zirbus, UK) for 48 h at − 83 ℃ to remove the moisture. Dried samples were sealed in plastic bags and stored in a refrigerator at 4 ℃ for further analysis in the laboratory.

### Preparation of seaweed extract

Dried seaweed sample was grounded to make fine powder as the finer the powder, the more efficient the extraction would be. Four gram of seaweed fine powder was soaked in 100 mL of solvent (water, methanol and ethanol) by maceration for the preparation of an extract by solvent extraction. The sample was kept in the dark for 24 h with intermittent shaking for better extraction. After incubation, the solution was filtered with Whatman filter paper No 4 (20–25 µm) retaining hygienic conditions. After filtration, the remaining wet powder was again extracted in their respective solvents for 12 h through sporadic shaking and filtered to get the maximum out of the sample powder. The methanol and ethanol extracts were then concentrated using a Rotary Vacuum Evaporator (LRE-702A, Labocon, UK) and Nitrogen Evaporator (AT-EV-50, Athena Technology, India) at 36 °C and the water solvent was dried by the Freeze Dryer (VaCo 2, Zirbus, UK) at − 83 ℃^16^. Finally working solutions were prepared as 1 mg mL^−1^, 3 mg mL^−1^, 5 mg mL^−1^ and 7 mg mL^−1^ for each extract.

### Qualitative analysis of phytochemical substances

Newly prepared all crude extracts of seaweed were subjected to qualitative assessments for the identification of various classes of active phytochemical constituents such as saponin^58^, terpenoid^59^, cardiac glycosides^60^ and phlobatannin^16^ following standard methods. General reactions in these analyses exposed the presence or absence of these compounds in the crude extracts tested.

### FTIR spectroscopy

Different crude extracts of *P. tetrastromatica* and *G. tenuistipitata* were used to determine the presence of characteristic peaks and their functional groups using FTIR spectroscopy (Perkin Elmer Spectrum 2)^61–63^. FTIR spectra were recorded within the wavelengths of 450 and 4000 cm^–1^. Analysis was done in triplicate and confirmed the spectrum in case of all extracts.

### Quantitative analysis of phytochemicals

#### Total phenolic content (TPC)

This parameter was carried out in the crude extracts using Folin-Ciocalteu Phenol reagents and external calibration with Gallic acid following by^41^ with slight modification. Briefly, 0.5 mL extract solution was added with 0.1 mL of FC reagent solution. After 15 min, 2.5 mL of saturated Na_2_CO_3_ (75 g L^−1^) was added in the solution and allowed to stand for 30 min at RT and absorbance was measured at 760 nm using the spectrophotometer (T80 + UV/Vis Spectrophotometer, UK). The concentration of total phenolics was calculated as mg of Gallic acid equivalent per gram. The calibration equation for Gallic acid was

1 $$ {\text{Y}} = 0.0116{\text{X}} + 0.0162;\quad {\text{R}}^{2} = 0.9987 $$

#### Total flavonoid content (TFC)

This parameter was computed in the crude extracts using the aluminum chloride colorimetric method with minor modifications^43^. Briefly, 1 mL extract solution was mixed with 3 mL methanol, 0.2 mL 10% aluminum chloride and 0.2 mL 1 M potassium acetate. The solution was then incubated at RT for 30 min and absorbance was measured at 420 nm. The concentration of total flavonoids was calculated as mg of quercetin equivalent per gram. The calibration equation for Quercetin was

2 $$ {\text{Y}} = 0.0102{\text{X}} - 0.0637;\quad {\text{R}}^{2} = 0.9693 $$

### Evaluation of total antioxidant capacity

#### DPPH (2, 2-diphenyl-1-picrylhydrazyl) assay

The DPPH free-radical scavenging assay was carried out in triplicate with negligible modification^21^. Different concentrated (1, 3, 5, 7 mg ml^−1^) aliquot extracts solution was mixed with 2.5 mL 0.15 mM DPPH solution (prepared in ethanol) and vortexes well. After 30 min incubation in dark, the absorbance of the mixture was read at 517 nm using spectrophotometer (T80 + UV/Vis Spectrophotometer, UK). Different concentrations were tested for each sample to get IC_50_ value which is defined as the amount of antioxidant necessary to decrease the initial DPPH ion by 50%. Ascorbic acid was used as a positive control. The percent radical scavenging activity of the crude extracts was calculated using the following formula:

3 $$ {\text{DPPH}}\;{\text{radical}}\;{\text{scavenging}}\;{\text{activity}}\;\left( \% \right) = \left[ {\left( {{\text{A}}_{0} {-}{\text{A}}_{{1}} } \right)/\left( {{\text{A}}_{0} } \right)} \right] \times {1}00 $$

where: A_0_ is the absorbance of DPPH radicals + methanol and A_1_ is the absorbance of DPPH radicals + sample extract.

#### ABTS radical scavenging assay

The antioxidant activities of different extracts were evaluated through the ABTS radical scavenging by the extracts ability to scavenge ABTS with slight modification^49^. Aliquot concentrations (1, 3, 5 and 7 mg mL^−1^) of extracts (50 µL) was added with 950 µL of ABTS solution (7 mM ABTS solution and 2.45 mM Potassium persulfate) followed by incubation at RT for 16 h in dark. Spectrophotometer (T80 + UV/Vis Spectrophotometer, UK) was applied to evaluate the absorbance at 734 nm. IC_50_ values were tested for each sample at each concentration. Ascorbic acid was used as a positive control. The percentage of inhibition was calculated using the following formula,

4 $$ {\text{ABTS}}\quad {\text{scavenged}}\left( \% \right) = \left[ {\left( {{\text{A}}_{{{\text{control}}}} {-}{\text{A}}_{{{\text{sample}}}} } \right)/\left( {{\text{A}}_{{{\text{control}}}} } \right)} \right] \times {1}00 $$

where: A_control_ is the absorbance of ABTS radicals + solvent and A_sample_ is the absorbance of ABTS radicals + sample extract.

#### Reducing power assay

Antioxidant activity of different crude extracts reducing power at various concentrations with insignificant modification^16^. Briefly, 1.5 µL of extracts was mixed with 1.5 µL of phosphate buffer (0.2 M, pH 6.6) and 1.5 µL of potassium hexacyanoferrate (1%, w/v). After incubation at 50 °C in a water bath for 20 min, 1.5 µL of trichloroacetic acid solution (10%) was added and centrifuge at 800×*g* for 10 min. The supernatant was collected and mixed with 3 mL of DW and 200 µL of ferric chloride solution (0.1%, w/v) and incubated at RT for 10 min for stable absorbance at 700 nm; as the more absorbance of the reaction mixture more the reducing power of the extracts will be. Here ascorbic acid was used as a positive control. Antioxidant activity was also expressed as equivalents of ascorbic acid.

#### Phosphomolybdenum assay

The antioxidant activity of different extract solution (water, ethanol and methanol) was evaluated by the green phosphomolybdenum complex formation with slight modification^15^. A reagent solution was prepared with 0.6 M H_2_SO_4_, 28 mM Sodium phosphate and 4 mM Ammonium molybdate. Further, 1.8 mL reagent solution was mixed with 0.2 mL of dilute extract solution and placed in a boiling water bath for 90 min at 95 °C. After cooling down, the absorbance of each sample was measured at 695 nm using spectrophotometer (T80 + UV/Vis Spectrophotometer, UK). Blank was run same procedure just replacing the extract with the equivalent solvent. Antioxidant activity was also expressed as equivalents of ascorbic acid.

#### Hydrogen peroxide scavenging activity

Extracts antioxidant activities were evaluated by the hydrogen peroxide scavenging activity with slight modification^28^. Briefly, aliquot extracts at various concentrations was added 0.3 mL hydrogen peroxide solution (40 mM) and 1.2 mL phosphate buffer (40 mM; pH 7.4) and vortexes well. Different concentrations were tested for each sample to get IC_50_ value. Ascorbic acid was used as a positive control. The percentage of inhibition of the crude extracts was calculated using the following formula:

5 $$ {\text{Hydrogen}}\;{\text{peroxide}}\;{\text{scavenged}}\left( \% \right) = \left[ {\left( {{\text{A}}_{0} {-}{\text{A}}_{{1}} } \right)/\left( {{\text{A}}_{0} } \right)} \right] \times {1}00 $$

where: A_0_ is the Absorbance of control and A_1_ is the Absorbance of sample solution.

#### NO scavenging assay

Different crude extracts antioxidant activity was evaluated by the NO scavenging ability with slight modification^14^. Briefly, 50 µL of extracts was mixed with 450 µL of sodium nitroprusside (SNP, 10 mM) and incubated the mixture at room temperature for 4 h. Further, 450 µL of griess reagent was added to the mixtures. After 10 min the absorbance was measured at 546 nm using spectrophotometer (T80 + UV/Vis Spectrophotometer, UK). IC_50_ values were tested for each sample at each concentration. Ascorbic acid was used as a positive control. The percentage of inhibition was calculated using the following formula,

6 $$ {\text{NO}}\quad {\text{scavenged}}\;\left( \% \right) = \left[ {\left( {{\text{A}}_{0} {-}{\text{A}}_{{1}} } \right)/\left( {{\text{A}}_{0} } \right)} \right] \times {1}00 $$

where: A_0_ is the Absorbance of control and A_1_ is the Absorbance of sample solution.

### Statistical analysis

The obtained experimental data was analyzed through the standard statistical procedure. Data were analyzed using SPSS software (IBM Co., Chicago, IL). Analysis of variance (ANOVA) and Duncan’s multiple range method were used to compare solvents and samples. Values were expressed as means ± standard deviations. Differences were considered significant at p < 0.05. All analyses were performed in triplicate.
